# Comparative margins between colorectal carcinoma and peritumoral injected methylene blue

**DOI:** 10.1590/0102-672020260000023e1952

**Published:** 2026-08-21

**Authors:** Priscila Oliveira CARDOSO, Andy PETROIANU

**Affiliations:** 1 Universidade Federal de Minas Gerais, Faculty of Medicine, Department of Surgery – Belo Horizonte (MG), Brazil.

**Keywords:** Colorectal Neoplasms, Hereditary Nonpolyposis, Margins of Excision, Methylene Blue, Lymphatic Metastasis, Colectomy, Neoplasias Colorretais, Hereditárias sem Polipose, Margens de Excisão, Azul de Metileno, Metástase Linfática, Colectomia

## Abstract

**Background:**

Removal of malignant tumors with free margins is pivotal in oncological surgery.

**Aims:**

To verify the correspondence between the histological growth of colon and rectal adenocarcinomas and how extensively methylene blue diffuses when injected into peritumoral mucosal tissue to understand if the dye margin can guide the correct margin for tumor removal, and also if there is an association between the lymph nodes stained by methylene blue and the presence of metastases.

**Methods:**

This study was conducted with 13 patients with colon or rectal adenocarcinoma. Immediately before the operation, all patients underwent colonoscopy and peritumoral methylene blue injection. Radical resection consisted of the removal of the colon or rectum segment beyond the blue-stained margins, in a monobloc, with the meso and regional lymph nodes. Tumor margins, peritumoral lymphatic density, stained margins, and removed lymph nodes were analyzed.

**Results:**

In all operative specimens, tumor-free margins were within the blue-stained area. There was no association between the presence of metastases and the dye in the lymph nodes examined.

**Conclusions:**

Preoperative peritumoral endoscopic injection of methylene blue spreads the dye beyond the limits of colon and rectal adenocarcinomas, determining reliable free margins for tumor resection, but does not indicate the presence of regional lymph node metastases.

## ARTICLE HIGHLIGHTS

Removal of small non-palpable tumors with free margins may be difficult, mainly in mini-invasive surgeries.

Preoperative peritumoral endoscopic injection of methylene blue spreads the dye beyond the limits of colon and rectal adenocarcinomas.

Methylene blue can be identified outside the colon and rectum, determining reliable free margins for tumor resection.

Methylene blue does not indicate the presence of regional lymph node metastases.

## Summary

**CENTRAL MESSAGE:**

Surgical treatment of colon and rectal adenocarcinomas should include the monobloc resection of the tumor with free margins, the mesorectum and mesocolon, all peritumoral tissue, and the regional lymph nodes up to the para-aortic and iliac ones. The microscopic growth of the tumor in the colonic wall usually reaches up to four centimeters. Therefore, tumor resection margins are considered complete when they extend more than five centimeters on macroscopic inspection. Dye injection into the peritumoral colonic or rectal mucosa spreads methylene blue through the colon and rectum beyond the microscopic extent of the cancer.

**PERSPECTIVES:**

Preoperative peritumoral endoscopic injection of methylene blue spreads the dye beyond the limits of colon and rectal adenocarcinomas, determining reliable free margins for tumor resection, but does not indicate the presence of regional lymph node metastases.

## VISUAL ABSTRACT

**Figure 2:**
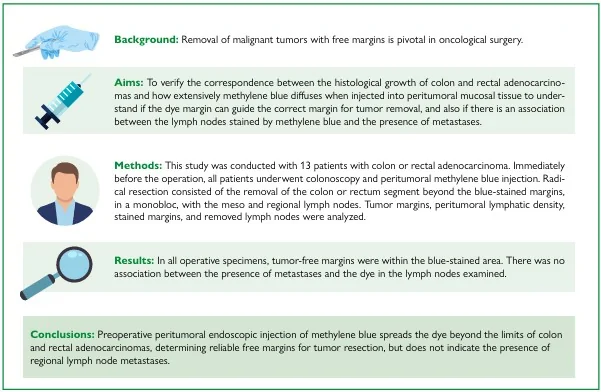

## RESUMO

**Racional:**

A remoção de tumores malignos com margens de segurança livres de neoplasia é fundamental nas operações oncológicas.

**Objetivos:**

Verificar se há correspondência entre o crescimento histológico do adenocarcinoma de cólon e de retal e a extensão de infiltração do azul de metileno injetado no tecido da mucosa peritumoral, a fim de saber se a margem do corante pode orientar a margem correta de remoção do tumor, e também se existe associação entre os linfonodos corados por azul e a presença de metástases.

**Métodos:**

Este estudo foi realizado em treze pacientes com adenocarcinoma de cólon ou reto. Imediatamente antes da operação, todos os pacientes foram submetidos a colonoscopia e injeção de azul de metileno peritumoral. A ressecção radical consistiu na remoção do segmento do cólon ou reto para além das margens azuis, em monobloco, incluindo os linfonodos do meso e regionais. Foram analisadas as margens tumorais, a densidade linfática peritumoral, as margens coradas e os linfonodos removidos.

**Resultados:**

Em todas as amostras operatórias, as margens sem tumor estavam dentro da área corada em azul. Não houve associação entre a presença de metástases e do corante nos linfonodos examinados.

**Conclusões:**

A injeção endoscópica peritumoral pré-operatória de azul de metileno difunde o corante para além dos limites do adenocarcinoma de cólon e de reto, determinando margens livres confiáveis para a ressecção tumoral, mas não indica a presença de metástases nos linfonodos regionais.

## INTRODUCTION

 Surgical treatment of colon and rectal adenocarcinomas should include the monobloc resection of the tumor with free margins, the mesorectum and mesocolon, all peritumoral tissue, and the regional lymph nodes up to the para-aortic and iliac ones^2,6,16^. In transverse colon tumors, the greater omentum must also be removed^1^. Determining cancer-free margins is essential to prevent residual tumor and protect the patient’s chance of cure^5^. 

 The microscopic growth of the tumor in the colonic wall usually reaches up to four centimeters. Therefore, tumor resection margins are considered complete when they extend more than five centimeters on macroscopic inspection^5,26,27^. In rectal cancers, the proximal margin must also be wide, but distally, tumor growth may be smaller because of the changing tissue from rectal mucosa to anal squamous epithelium^2,13,18,21^. The Current National Comprehensive Cancer Network Guideline recommends a circumferential margin of five centimeters for total mesorectal excision and two centimeters for the distal margin in low rectal tumors^10^. With the progression of minimally invasive surgery, with or without robotic assistance, identifying tumor margins by palpation is difficult. Therefore, tumor dimensions must be visually established for a precise oncological surgical procedure^4,8,23^. 

 Dye injection into the colonic or peritumoral rectal mucosa can spread through the colon up to a certain distance, which can be associated with the microscopic extent of the cancer^11,16,25^. Therefore, detecting the dye externally from the colon can guide the level of tumor resection. In this sense, this study aimed to verify the correspondence between the histological growth of colon and rectal adenocarcinomas and how extensively methylene blue diffuses when injected into the peritumoral mucosal tissue to understand if the dye margin can guide the correct margin for tumor removal, and also if there is an association between the lymph nodes stained by methylene blue and the presence of metastases. 

## METHODS

 This research was approved by the Research Ethics Committee of the Institution under number 937038. All patients were invited to participate in the study, and were included only after they had agreed and signed the Informed Consent Form. 

 The study was performed on 13 patients, nine men and four women, aged 36 to 82 years (64.5±15.0) with a diagnosis of adenocarcinoma of the descending colon (3 patients), sigmoid (5 patients), and rectum (5 patients). All patients had preoperative confirmation of adenocarcinoma by colonoscopic biopsy and histological exam. 

**Figure 1:**
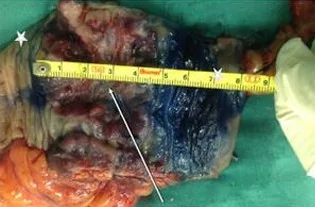
Image of sigmoid segment containing adenocarcinoma [arrow]. Note the methylene blue that was injected peritumorally immediately before surgery, delimiting the resection margins [*].

 Immediately before starting the surgery, in the operating room, a colonoscopy was performed, and 5 ml of methylene blue was injected into the mucosa circumferentially around the entire tumor. Then, the tumor was removed in monobloc with surgical margins encompassing the entire tissue stained in blue, along with the mesocolon and mesorectum, peritumoral tissue, and regional lymph nodes up to the origin of the colon and rectum arteries from the iliac artery and aorta. The operations were performed through laparoscopy in seven patients and laparotomy in the other six patients. The surgical specimen was opened, and it was confirmed that the removal of the colon and rectum included the macroscopic limits of the tumor and all methylene blue margins. Margins on the fresh surgical specimen were measured in millimeters, using a millimeter ruler (Figure 1). 

 The monobloc surgical specimen was fixed in 10% buffered formaldehyde solution, processed according to the anatomopathological routine, and stained with hematoxylin and eosin. A single pathologist conducted the histological examination. The histological margins of the cancer were recorded, considering its depth, angiolymphatic and perineural invasion, area of tumor necrosis, tumor budding (islets of undifferentiated tumor cells around the larger tumor), intra and peritumoral lymphocyte infiltrate, and the presence of lymph node metastases. The distal and proximal margins of the tumor were analyzed by immunohistochemistry with anti-D2-40 antibody. The density of lymphatic tissue markers was quantified from the tumor in the proximal and distal tumor margins. To assess the density of peritumoral lymphatic tissue, the area with the highest lymphatic density was chosen by D2-40 (hot spots) with a 40X objective. Then, the vessels were counted, extending the count to adjacent fields, until an area of 1 mm² was reached. A 400X objective was used. All the microscopic limits of colon or rectal adenocarcinoma were compared with the macroscopic limits of methylene blue in the surgical specimen. Each lymph node was processed, and the presence of metastases was established in the histological sections. Lymph nodes with metastases and all blue-stained lymph nodes were recorded. 

 For the statistical analysis, the Statistical Package for Social Sciences version 20.0 for Windows, SPSS Incorporation, Chicago, Illinois, United States of America, and Microsoft Office Excel 2007 were used. A descriptive analysis of the variables used in the study was performed, and qualitative or categorical variables were tabulated for frequency distribution. A t-test for paired samples was used to assess global lymphatic density in relation to the pathological stage of the colon neoplasm. Parametric continuous variables were presented as means and standard deviation of means. The difference corresponding to p<0.05 was considered significant. 

## RESULTS

 Tumor staging after surgery and pathological examination was I (three patients), IIA (seven patients), IIIB (one patient), and IVA (two patients). Histologically, the adenocarcinoma was intestinal (12 patients) and mucinous (1 patient). Peritumoral lymphocytic infiltrate was present in ten patients. Vascular and perineural invasion was found in only one patient. Invasion of the colorectal wall reached the muscular layer in three patients, the serous layer in one patient, and pericolonic fat in nine patients. More than 12 lymph nodes were present in the surgical specimens. In three patients, metastases were found in three, three, and two lymph nodes, respectively. 

 All surgical specimens had tumor-free margins within the methylene blue area, which extended to 50 mm. Neoplastic cells were found up to 20 mm distally and 15 mm proximally beyond the macroscopic margins of the adenocarcinomas. The density of peritumoral lymphatic tissue reached up to about 40 mm distally and 35 mm proximally to the macroscopic dimensions of the tumors. Therefore, more than 10 mm beyond the histological limits of the tumors are within the methylene blue-stained area. The margins outlined by the dye were more extensive than the histological and peritumoral lymphatic density margins in all cases. 

 Methylene blue was present in several lymph nodes without metastases, but not in all of them. The dye was found in six of the eight lymph nodes with metastases. Some lymph nodes were stained on the inside, but the blue color was not visible on the outside. Therefore, no association between the presence of metastasis and the dye was found, and the presence of the dye inside a lymph node did not necessarily mean it was visible from the outside. 

## DISCUSSION

 Removing the tumor with clear margins is a fundamental oncologic principle in curative-intent surgeries and determines the complementary postoperative procedures, indicating prognosis, morbidity, and mortality^2,5,6,16,26^. In addition to tumor, node, and metastasis (TNM) staging, there are several histological aspects to consider about tumor margins, including tumor budding, angiolymphatic infiltration, perineural invasion, and tumor and peritumoral lymphangiogenesis^1,3,12,14,17,22,24,27^. 

 In colon and especially in rectal adenocarcinomas, the extension of the distal margin is a controversial topic in the literature^5,16,19,21^. Intramural distal growth of the rectal tumor rarely exceeds one to two centimeters and does not compromise local control or survival. Cipe et al. verified the distal tumor margin by injecting carbon particles through colonoscopy before surgery^7^. The Current National Comprehensive Cancer Network Guideline recommends a circumferential margin of four to five centimeters for total mesorectal excision and one to two centimeters for the distal margin in the low rectal tumor^10^. Lin and Pollard evaluated 148 resected rectal cancers with a distal margin smaller than one centimeter and found no local recurrence^15^. Cross et al. considered the microscopic extension of the tumor margin irrelevant as long as the macroscopic margin is two centimeters, with angiolymphatic invasion being the most important data^9^. 

 Elevated peritumoral lymphatic vessel density is associated with a worse prognosis even in stage I colorectal adenocarcinomas^3,14 17,23,24^. Lymphangiogenesis is crucial for the prognosis of tumors in early stages, but not in advanced disease^3,5 12,14^. Lymphatic vessels are one of the main routes of tumor dissemination, which can occur through pre-existing and newly formed lymphatic vessels in and around the tumor. Adenocarcinoma cells release growth factors from lymphatic endothelium, inducing enlargement of existing lymphatic vessels, proliferation of endothelial cells, and facilitating tumor cell migration and metastatic spread^5,14^. Therefore, intra and peritumoral lymphatic density must be measured through an immunohistochemical study, so that this tissue is also included in the surgical specimen, thus reducing the risk of metastases or residual local neoplastic islands^1,16 26,27^. In this study, no significant increase in lymphatic density was identified in the tissues removed, and intratumoral lymphatic vessel density was lower than peritumoral lymphatic vessel density. No evidence of neoplastic cells or microscopic intramural dissemination beyond four centimeters was found, whereas methylene blue staining was extended to five centimeters. Therefore, the resection of the colon and rectum at the margins of blue staining ensures the complete removal of the tumor, including its microscopic lymphatic dissemination. 

 Despite the small number of cases, the extent of the blue staining in the colonic and rectal wall beyond the histological margins of the tumor and peritumoral lymphatic tissue in all cases provides statistical support for the reliability of the results. Peritumoral dye injection facilitated surgical guidance both in laparoscopy and laparotomy for tumor resection following oncological principles^7,11 18,20^. Tumor resection guided by the visualized methylene blue avoids the need to delimit the tumor by palpation, which is subjective and hard to be precise in minimally invasive surgeries, including those performed with robotic assistance. 

 On the other hand, methylene blue is not a good indicator of lymph node metastases as it does not stain all lymph nodes with metastases^20^. Even internally stained lymph nodes may not show this staining on the outside, which would otherwise be visible during surgical procedures. Therefore, methylene blue staining is not associated with lymph node metastases and is not useful to indicate the possibility of regional spread of colon and rectal adenocarcinoma. This finding should be strongly and carefully considered in procedures based on sentinel lymph nodes by injecting a radioactive solution, blue dye, and performing a biopsy as signs and tests of cancer spread beyond the original tumor. Positive results are valid, but negative ones are not. 

 Thus, preoperative peritumoral endoscopic injection of methylene blue spreads the dye beyond the limits of colon and rectal adenocarcinomas. Methylene blue can be identified outside these organs, determining reliable free margins for tumor resection, but it does not indicate the presence of regional lymph node metastases. This method can be useful before surgical resection of other digestive tumors, such as esophageal, gastric, duodenal, and even jejuno-ileal tumors, through minimally invasive surgeries. Removal of the entire methylene blue-stained tissue reduces the possibility of tumor resection with insufficient cancer-free margins. 

## CONCLUSIONS

 Preoperative peritumoral endoscopic injection of methylene blue spreads the dye beyond the limits of colon and rectal adenocarcinomas, determining reliable free margins for tumor resection. However, it does not indicate the presence of regional lymph node metastases. 

## Data Availability

The datasets generated and/or analyzed during the current study are available from the corresponding author upon reasonable request.

## References

[1] Ahmad NZ, Azam M, Fraser CN, Coffey JC (2023). A systematic review and meta-analysis of the use of methylene blue to improve the lymph node harvest in rectal cancer surgery. Tech Coloproctol.

[2] Andreola S, Leo E, Belli F, Lavarino C, Bufalino R, Tomasic G (1997). Distal intramural spread in adenocarcinoma of the lower third of the rectum treated with total rectal resection and coloanal anastomosis. Dis Colon Rectum.

[3] Barresi V, Reggiani-Bonetti L, Di Gregorio G, Ponz De Leon M, Barresi G (2011). Lymphatic vessel density and its prognostic value in stage I colorectal carcinoma. J Clin Pathol.

[4] Burghgraef TA, Crolla RMPH, Verheijen PM, Fahim M, van Geloven A, Leijtens JWA (2022). Robot-assisted total mesorectal excision versus laparoscopic total mesorectal excision: a retrospective propensity score-matched cohort analysis in experienced centers. Dis Colon Rectum.

[5] Capobiango A, Araújo ID, Petroianu A (1992). Estudo epidemiológico das neoplasias malignas do intestino grosso e anus no Estado de Minas Gerais. Rev Bras Colo-Proctol.

[6] Carvalho A, Limbert M, Cabral F, Fareleira A, Duarte A, Barroca R (2024). The impact of methylene blue in colon cancer: a retrospective multicentric study. Int J Colorectal Dis.

[7] Cipe G, Cengiz MB, Idiz UO, Yardimci E, Malya U, Firat D (2016). The effects of preoperative endoscopic tattooing on distal surgical margin and ileostomy rates in laparoscopic rectal cancer surgery: a prospective randomized study. Surg Laparosc Endosc Percutan Tech.

[8] Çakır T, Aslaner A (2021). Early results of novel robotic surgery-assisted low anterior resection for rectal cancer and transvaginal specimen extraction by using Da Vinci XI: initial clinical experience. Rev Assoc Med Bras (1992).

[9] Cross SS, Bull AD, Smith JH (1989). Is there any justification for the routine examination of bowel resection margins in colorectal adenocarcinoma?. J Clin Pathol.

[10] Desai AP, Chengappa M, Go RS, Poonacha TK (2020). Financial conflicts of interest among National Comprehensive Cancer Network clinical practice guideline panelists in 2019. Cancer.

[11] Feo CV, Portinari M, Zuolo M, Targa S, Matarese VG, Gafà R (2015). Preoperative endoscopic tattooing to mark the tumour site does not improve lymph node retrieval in colorectal cancer: a retrospective cohort study. J Negat Results Biomed.

[12] Gao J, Knutsen A, Arbman G, Carstensen J, Frånlund B, Sun XF (2009). Clinical and biological significance of angiogenesis and lymphangiogenesis in colorectal cancer. Dig Liver Dis.

[13] Ghersin I, Sroka G, Haj B, Ghersin DS, Matter I (2014). Inadvertent tattooing of adjacent large bowel: a case report and review of literature. ABCD Arq Bras Cir Dig.

[14] Li LR, Fang YJ, Pan ZZ, Wu XJ, Wan DS, Hardingham JE (2010). Length of lymphangiogenesis in the rectal tissues distal to rectal cancer. Tumour Biol.

[15] Lin EY, Pollard JW (2004). Role of infiltrated leucocytes in tumour growth and spread. Br J Cancer.

[16] Maguire A, Sheahan K (2014). Controversies in the pathological assessment of colorectal cancer. World J Gastroenterol.

[17] Nagahashi M, Ramachandran S, Rashid OM, Takabe K (2010). Lymphangiogenesis: a new player in cancer progression. World J Gastroenterol.

[18] Norouzi S, Alipour E, Soltani S, Hallaj-Nezhadi S, Hashemzadeh S (2025). A novel electrochemical biosensor with liposomal amplification for sensitive detection of colon cancer biomarker: a step toward early cancer diagnosis. Talanta.

[19] Park IJ, Kim JC (2010). Adequate length of the distal resection margin in rectal cancer: from the oncological point of view. J Gastrointest Surg.

[20] Park JS, Chang IT, Park SJ, Kim BG, Choi YS, Cha SJ (2009). Comparison of ex vivo and in vivo injection of blue dye in sentinel lymph node mapping for colorectal cancer. World J Surg.

[21] Pricolo VE, Abodeely A, Resnick M (2010). Distal margins in radical resections for rectal cancer after chemoradiation therapy: how short is long enough?. Dig Surg.

[22] Santos EKAN, Triches BG, Silva GP, Linhares JC, Mehanna SH, Cavalcanti MS (2025). Peritumoral budding as a predictor for lymph node metastases in colorectal carcinomas: what is the importance?. ABCD Arq Bras Cir Dig.

[23] Somashekhar SP, Reddy GRK, Deshpande AY, Ashwin KR, Kumar R (2021). A prospective study of real-time identification of line of transection in robotic colorectal cancer surgery by ICG. J Robot Surg.

[24] Stacker SA, Williams SP, Karnezis T, Shayan R, Fox SB, Achen MG (2014). Lymphangiogenesis and lymphatic vessel remodelling in cancer. Nat Rev Cancer.

[25] Staniloaie D, Budin C, Vasile D, Iancu G, Ilco A, Voiculescu DI (2022). Role of methylene blue in detecting the sentinel lymph node in colorectal cancer: in vivo vs. ex vivo technique. Exp Ther Med.

[26] Ueno H, Shirouzu K, Eishi Y, Yamada K, Kusumi T, Kushima R (2013). Characterization of perineural invasion as a component of colorectal cancer staging. Am J Surg Pathol.

[27] Ueno H, Shirouzu K, Shimazaki H, Kawachi H, Eishi Y, Ajioka Y (2014). Histogenesis and prognostic value of myenteric spread in colorectal cancer: a Japanese multiinstitutional study. J Gastroenterol.

